# Terahertz Time-Domain Polarimetry in Reflection for Film Characterization

**DOI:** 10.3390/s20123352

**Published:** 2020-06-12

**Authors:** Sandrine van Frank, Elisabeth Leiss-Holzinger, Michael Pfleger, Christian Rankl

**Affiliations:** Research Center for Non-Destructive Testing GmbH (RECENDT), Altenberger Straße 69, 4040 Linz, Austria; sandrine.vanfrank@recendt.at (S.v.F.); elisabeth.leiss-holzinger@recendt.at (E.L.-H.); michael.pfleger@recendt.at (M.P.)

**Keywords:** terahertz, time-domain spectroscopy, polarimetry, film characterization, brewster angle, thickness

## Abstract

Terahertz time-domain spectroscopy is a useful technique to characterize layered samples and thin films. It gives access to their optical properties and thickness. Such measurements are done in transmission, which requires access to the sample from opposite sides. In reality this is not always possible. In such cases, reflection measurements are the only option, but they are more difficult to implement. Here we propose a method to characterize films in reflection geometry using a polarimetric approach based on the identification of Brewster angle and modeling of the measured signal to extract the refractive index and thickness of the sample. The technique is demonstrated experimentally on an unsupported single layer thin film sample. The extracted optical properties and thickness were in good agreement with established transmission terahertz spectroscopy measurements. The new method has the potential to cover a wide range of applications, both for research and industrial purposes.

## 1. Introduction

Terahertz time-domain (THz-TDS) spectroscopy [1] has been a topic of research for more than 10 years [2] and has proved efficient to characterize the optical parameters of a wide variety of materials [3,4,5,6,7]. This technique is commonly applied in laboratories to characterize or identify samples and is now finding its way into industrial applications [8,9,10]. Up to now it has mostly been applied to measurements in transmission geometry. Although this arrangement is practical in a laboratory, it imposes a number of limitations on the samples in practical applications. The strictest requirement is that the sample has to be at least partially transparent for THz radiation. This means that it cannot be too thick or too opaque, or placed on top of other non-transparent materials such as metals. Even if a transparent substrate can be used, it needs to be characterized independently. These restrictions can be lifted by carrying out spectroscopy measurements in reflection geometry and some methods have been investigated to characterize materials and films in reflection [11,12,13,14,15,16,17,18]. However, reflection measurements present their own difficulties when it comes to evaluating optical parameters, mostly due to the necessity of a very precise positioning of the samples [12,13,19]. Techniques common in other frequency ranges, such as ellipsometry [20], may be tricky to implement for pulsed THz due to the scarcity of adapted components (e.g., quarter waveplate or broadband polarizer).

Here we propose a polarimetric approach to reflection THz-TDS based on a simple flexible THz-TDS setup to estimate the optical parameters and thickness of thin material layers or films. The analysis relies on the simulation of the Fresnel reflection coefficients and the measurement of the Brewster angle [21,22,23,24] to evaluate the frequency-dependent refractive index of the material and of the pulse retardation to extract the layer’s thickness. In the present paper we demonstrate this approach in the case of an unsupported single layer film. Examples of applications are membranes, lids or seals, but also layered materials with air gaps. In principle, this method can be extended to the more general case of a film on a substrate. This polarimetric approach was enabled by adding rotating stages for the THz emitter and detector combined with a precision reflection guide to a THz-TDS setup. Even with these additions the setup is simple and only consists of a few elements. It allows for an easy and accurate identification of the optimal measurement angles for the film characterization. In general, both the setup and the analysis are simple to implement in contrast to other reflection approaches.

## 2. Materials & Methods

### 2.1. Setup Configuration

**Figure 1:**
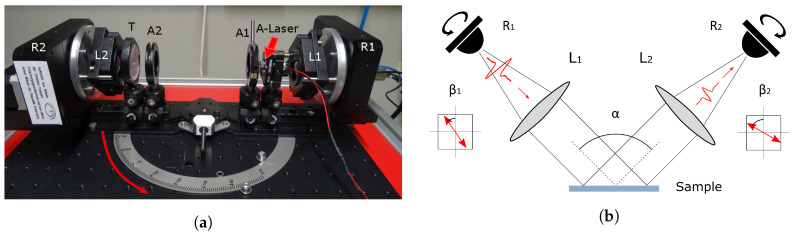
(**a**) Photography of the setup with the reflection guide open at $180^{\circ }$ and all the elements in place for the second step of the alignment procedure: fiber-based PCAs mounted in rotation stages ($R_{1}$, $R_{2}$), lenses for beam collimation ($L_{1}$, $L_{2}$), irises ($A_{1}$, $A_{2}$), aiming laser (*A*-Laser) and target (*T*). (**b**) Schematic representation of the setup after alignment, positioned for a measurement in reflection.

A terahertz time-domain spectrometer based on a fiber-laser in combination with standard fiber-coupled photo-conductive antennas (PCAs) from Menlo Systems GmbH (Tera15-FC) was used to emit and detect the THz beam (Figure 1a). The emission PCA is a strip-line antenna, while the detection PCA has a dipole geometry. The elements were mounted on a reflection guide, which allows for a precise adjustment of the incidence angle α (Figure 1b) of the THz beam on the sample between $70^{\circ }$ and $180^{\circ }$ (i.e., full opening for transmission measurements). The PCAs were integrated into self-designed holders equipped with rotation motors ($R_{1}$ and $R_{2}$), so that emitter and detector can be rotated independently of each other by a full $360^{\circ }$. As a result of its structure, the PCA source emits a strongly linear polarized THz beam [25] along a well-defined axis. Likewise, the PCA detector shows the highest sensitivity along the same axis. By rotating the PCAs, both the direction of polarization of the THz beam incident on the sample surface ($\beta_{1}$ in Figure 1b) and the preferred direction of detection ($\beta_{2}$) can be easily adjusted. The rotation stages include an adjustable mount for lenses ($L_{1}$ and $L_{2}$) that ensure a good alignment between the axes of the PCAs and the collimating lenses. The rotation sequence of the PCAs is completely automatized for the measurements.

### 2.2. Optics and Sample Alignments

The first step of the alignment is performed in transmission geometry ($\alpha =180^{\circ }$). The aim is to optimize the position of the optical elements to obtain rotation invariance of the signal when rotating the PCAs in the same direction ($\beta_{1}=\beta_{2}$). Here the simplicity of the setup (minimum number of elements, use of lenses instead of mirrors) offers a great advantage as the number of necessary adjustments is minimized. In order to reach a constant signal at the detector, the positions of the collimating lenses $L_{1}$ and $L_{2}$ are adjusted in the *x*, *y* and *z*-directions. Iris apertures ($A_{1}$ and $A_{2}$) were used to localize the beam center and direction adjusting $L_{1}$ and $L_{2}$ accordingly. The signal is optimized to show an optimal amplitude while keeping a constant shape for all $\beta_{1}=\beta_{2}$ and a quasi-null amplitude for $\beta_{1}=\beta_{2}+90^{\circ }$ (cross-polarization). This optical alignment needs to be carried out only once.

In a second step, an aiming laser (*A*-Laser) and target (*T*) are mounted on the rail of the reflection guide (Figure 1a). The laser is aligned until it hits *T* after passing both apertures $A_{1}$ and $A_{2}$. A sample holder containing either a mirror (in the case of a reference measurement) or a sample is then inserted in the center of the reflection guide. This holder consists of a goniometer and a linear stage with which the depth position and the two tilt angles of the sample can be modified. The angle of the reflection guide is then varied and the holder’s positioning parameters adjusted until the laser hits the *T* for all αs. This part of the alignment has to be checked again after mounting a new sample. Finally, the aiming laser and the target are removed and the position of the linear stage is fine-tuned using the THz signal until the travel time of the THz pulse stays constant for all αs. The setup ready for measurements is sketched in Figure 1b.

The success of the alignment procedure is verified using a reflection measurement on a mirror as a reference. The antennas are rotated and the measured THz signal is compared to the expected amplitude of the detected signal as a function of the PCA angles $\beta_{1},\beta_{2}$, assuming perfect linear polarization of the emitted THz beam, perfect linear detection and perfect alignment.

### 2.3. Polyvinyl Chloride Film

Experimental testing of our method was done using a Polyvinyl chloride film (PVC) sample. The sample was taken from a cover sheet of a regular office notepad. Using Mid infrared spectroscopy it was verified that this film was made of PVC (data not shown). The thickness was determined by optical coherence tomography (OCT) as well as mechanically by stacking 8 pieces of the sheet and measuring its total thickness by a caliper. The average determined thickness was 200±10μm and 202±20μm for caliper and OCT method, respectively.

### 2.4. Refractive Index Measurement

**Figure 2:**
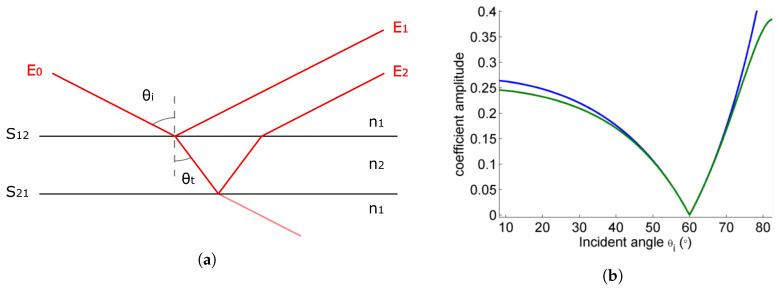
(**a**) Sketch of the film sample and the terahertz (THz) beam reflected or transmitted at the interfaces $S_{12}$ and $S_{21}$. (**b**) Reflection coefficients behavior for a THz beam reflected off the front ($S_{12}$, blue) and back ($S_{21}$, green) interfaces for arbitrary material parameters.

The approach chosen in this work to extract the refractive index of an unsupported film is based on the Fresnel equations and the Brewster angle principle. The THz beam impinging on the interface between two isotropic optical media is like any electromagnetic wave, described by the well-known Fresnel equations [26]:

(1) $$\begin{array}{cccc} r_{p} & =\frac{n_{t}\cos\left(\theta_{i}\right)-n_{i}\cos\left(\theta_{t}\right)}{n_{i}\cos\left(\theta_{t}\right)+n_{t}\cos\left(\theta_{i}\right)} & t_{p} & =\frac{2n_{i}\cos\left(\theta_{i}\right)}{n_{i}\cos\left(\theta_{t}\right)+n_{t}\cos\left(\theta_{i}\right)} \end{array}$$

(2) $$\begin{array}{cccc} r_{s} & =\frac{n_{i}\cos\left(\theta_{i}\right)-n_{t}\cos\left(\theta_{t}\right)}{n_{i}\cos\left(\theta_{i}\right)+n_{t}\cos\left(\theta_{t}\right)} & t_{s} & =\frac{2n_{i}\cos\left(\theta_{i}\right)}{n_{i}\cos\left(\theta_{i}\right)+n_{t}\cos\left(\theta_{t}\right)}, \end{array}$$

where *r* and *t* denote the reflection and transmission coefficients respectively, while *p* and *s* stand for parallel and perpendicular (“senkrecht”) polarizations, respectively; $\theta_{i}$ represents the incident angle of the light in the first medium with refractive index $n_{i}$, while $\theta_{t}$ is the angle of transmitted light in the second medium with refractive index $n_{t}$. This formulation applies to the case of a linear, homogeneous dielectric. In this case, the reflection coefficient $r_{p}$ goes through zero for a specific value of the incidence angle, called Brewster angle, which depends on the refractive indices of the media in presence according to $\tan\left(\theta_{B}\right)=n_{t}/n_{i}$. In the case of an unsupported single layer film, this angle is the same for the front and back surfaces of the sample (see Figure 2b).

The Brewster angle analysis is carried out on the frequency spectrum using a Fourier Transformation in order to extract the frequency-dependent refractive index of the material n(ν). The amplitude of the spectrum measured at different opening angles of the reflection guide are compared with numerical values obtained using Equation (1). A fit on the data is made using a parabolic fit method as proposed in [27] to obtain a precise measurement of the Brewster angle. The fit is conducted using the scaled reflection coefficient $R_{p}=r_{p}^{2}/\left(r_{p}^{2}+r_{s}^{2}\right)$ on the accordingly transformed amplitude data, which will be denoted $I_{p}$. For comparison, the Brewster angle obtained from the time-domain data is also extracted.

### 2.5. Film Thickness Evaluation

Once the refractive index of a film has been determined by the Brewster method, its thickness *d* is estimated using the same measurement procedure as applied for the reference measurement on the mirror, with an automated sequence for the rotation of the PCAs. The THz-TDS signal is composed of multiple pulses, where each reflected pulse is delayed with regard to the previous pulse by a time

(3) $$\Delta_{t}=2nd\cos\left(\theta_{t}\right)/c,$$

which in turn gives the thickness of the film

(4) $$d=\frac{c\Delta_{t}}{2n}/\sqrt{1-{\left(\sin\left(\theta_{i}\right)/n\right)}^{2}}.$$

The signal at the detector is described in the frequency domain as:

(5) $$E_{det}\left(\nu \right)=E_{1}\left(\nu \right)+E_{2}\left(\nu \right)=E_{0}\left(\nu \right)\times \left[r_{1}+r_{2}e^{-i2\pi \nu \Delta_{t}}\right],$$

where ν is the frequency, $E_{0}$ the input signal before reflection on the surface, $r_{1}$ the reflection coefficient on the first interface and $r_{2}$ the effective reflection coefficient on the backside including transmission through the first interface and $\Delta_{t}$ is the frequency-dependent time delay between first and second pulse. Additional reflections are ignored. Both reflection coefficients depend on the PCAs’ orientations. The input signal $E_{0}$ is determined by using a mirror as target directly after the alignment check procedure. This equation is used to model the signal propagation in the film and fit the data for various combinations of $\left(\beta_{1},\beta_{2}\right)$. Details of the calculation can be found in Appendix A.

### 2.6. Transmission Spectroscopy

In order to validate the refractive index obtained by the Brewster angle measurement method proposed in this paper, the sample was also measured using standard transmission THz spectroscopy. This is made possible by the fact that the film used for this demonstration can be detached and fixed on a different holder adapted to transmission measurements. The refractive index obtained with the Brewster method was compared to the transmission spectroscopy data analyzed with the software TeraLyzer [28,29]. This yields reference values for the frequency-dependent refractive index over a large frequency range as well as an estimation of the film thickness.

## 3. Results

### 3.1. Reflective THz-TDS Polarimetry Setup

**Figure 3:**
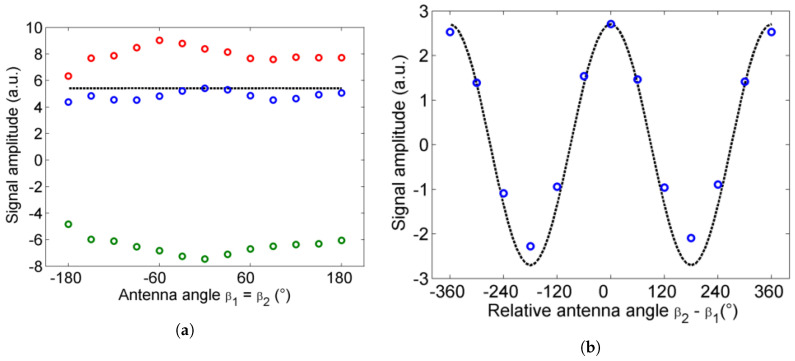
(**a**) Variations in the signal when rotating the PCAs in the same direction: pulse minimum (green), pulse maximum (red) and spectrum amplitude (blue) compared to the model (black). (**b**) Variations in the signal when rotating only the emitter (blue) compared to the model (black).

To the best of our knowledge, the proposed experimental setup (see Section 2.1) with its rotating antennas is the first of this type in the literature. The results of the alignment (see Section 2.2) are shown in Figure 3 as sub-vectors of a 13×13 matrix comprising data for as many $\left(\beta_{1},\beta_{2}\right)$ combinations corresponding to $30^{\circ }$ steps in the PCA’s rotation. After careful adjustment of the optics and the mirror with the PCAs’ axes aligned, the signal remains convincingly stable when both antennas are rotated in the same direction (Figure 3a). The deviation of the amplitude over the whole $360^{\circ }$ range reaches a maximum of 15% compared to the average value. When the PCAs are rotated in opposite directions, the signal amplitude follows a sine curve with a $360^{\circ }$ period, as expected from the relative orientation of their polarization axes (Figure 3b). A slight deviation from the model can be observed there. These deviations can be attributed to several effects. In particular imperfections of the PCA’s emission and/or detection axis play a clear role. Even if done perfectly, the collimating lens alignment cannot completely correct for the precession of the beam during rotation of the stage, which is due to a slight decentering of the THz emitter with respect to the rotation axis. Nevertheless, at the end of this simple alignment procedure it is possible to measure the refractive index and thickness of a film in reflection, as will be shown in the next sections.

### 3.2. Refractive Index

The refractive index of an unsupported single layer thin PVC film was assessed. For this the mirror used for the alignment was replaced by the film and the last alignment step was repeated to ensure that the position and tilt of the sample are correct. The incident angle α should then be equal to $2\theta_{i}$. The angle α was varied between $90^{\circ }$ and $135^{\circ }$ in steps of $2^{\circ }$. For each α one time trace was recorded for $\left(\beta_{1},\beta_{2}\right)=\left(0^{\circ },0^{\circ }\right)$ and $\left(\beta_{1},\beta_{2}\right)=\left(90^{\circ },90^{\circ }\right)$, which correspond to the *p*- and *s*-polarization, respectively. As Bahrim et al. [27] showed, only a few measurement angles before and after the critical angle $\alpha_{B}$ should be necessary to obtain a fit with good precision.

**Figure 4:**
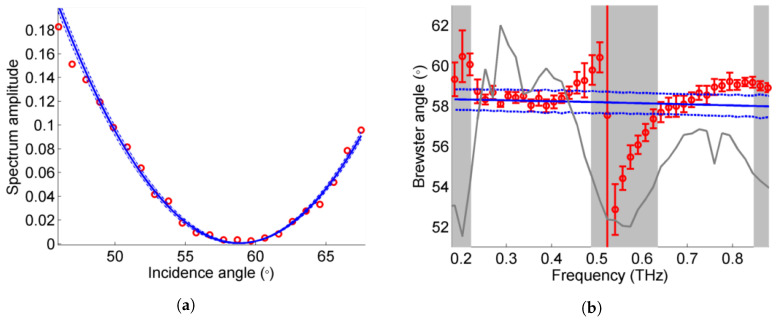
(**a**) Example of a fit on $I_{p}$ for ν=0.27 THz. (**b**) Brewster angle as a function of the frequency, compared with the results of the spectroscopy in transmission (blue lines); the spectrum (gray line) is shown for reference, the frequency ranges with low signal are darkened.

**Figure 5:**
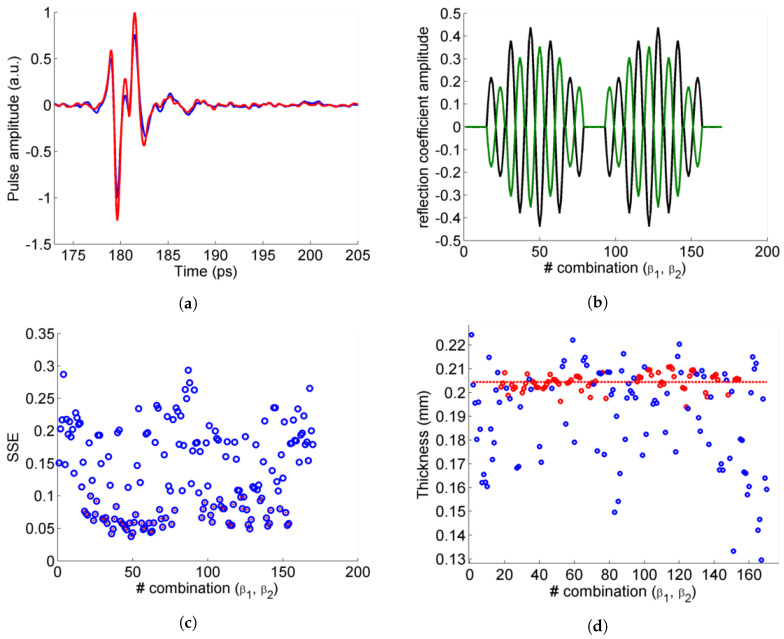
(**a**) Example of overlapping pulses in the experimental signal (blue) and corresponding fit (red). (**b**) Amplitude of reflection coefficients from first interface $r_{1}$ (black) and backside $r_{2}$ (green). (**c**) Sum of squared estimate of erros (SSE) for each fit (blue circles), selected points of low SSE (red dots). (**d**) Thickness obtained from the fits with high SSE (blue dots) and low SSE (red dots), as well as averaged thickness value (red line).

For any film, there is not one but multiple possibly overlapping reflections to account for, as illustrated in Figure 2a. However, unsupported single layer films present the particularity to have the same reflection coefficients (up to a minus sign) for the front and back side of the sample, as the medium on both sides of the sample is the same. The transmission coefficients, on the other hand, are not symmetric. However, because they get close to 1 for the *p*-polarization in the vicinity of the Brewster angle, the effective reflection coefficients for two pulses reflected on the first and second interfaces respectively are almost identical in a range of several tens of degrees (see Figure 2b). As expected, the measured signal amplitude as a function of incident angle has a convex shape (Figure 4a). Two pulses stemming from the reflections on the interfaces $S_{12}$ and $S_{21}$ dominate the signal and overlap slightly (see example in Figure 5a). Additional pulses coming from internal reflections within the film have negligible amplitude.

The PCAs used in the setup are pulsed and thus non-monochromatic broadband sources. With such sources, the Brewster angle analysis can be carried out on the pulses themselves (time-domain information) or on the Fourier spectrum (frequency-domain information). One advantage of the Fourier spectrum is the possibility to carry out the analysis for different frequencies and thus get frequency-dependent n(ν). The time-domain signals are thus Fourier-transformed and the amplitudes in the frequency-domain are transformed using $I_{p}=|E_{det,0^{\circ }}{|}^{2}/\left(\right|E_{det,0^{\circ }}{|}^{2}+|E_{det,90^{\circ }}{|}^{2})$. The transformed signal is then fitted with the parabolic function $R_{p}$ to find the Brewster angle $\alpha_{B}\left(\nu \right)$, as described in the method in Section 2.4. The result of the fit is shown in Figure 4. The results are only considered relevant in the regions with high enough spectrum amplitude, here in the ranges 0.2–0.5 THz and 0.6–0.9 THz (see gray line and shaded regions in Figure 4a). The dip between 0.5–0.6 THz corresponds to an oscillation in the spectrum due to the presence of more than one reflected pulse in the time-domain signal, also called Fabry–Perot oscillation.

The refractive index $n_{FD}\left(\nu \right)$ deduced from the Brewster angles estimated for different frequencies lies in the range [1.602−1.641] for the highest spectrum amplitudes, i.e., between 0.25 and 0.45 THz, with an uncertainty <0.022 (95% confidence interval of the fit). The averaged refractive index on this interval is ${\bar{n}}_{FD}=1.614$, which agrees very well with the averaged value found by transmission spectroscopy on this interval ${\bar{n}}_{transm.,0.25-0.45}=1.617\pm 0.014$. For comparison, the Brewster angle estimation using the same fit method is also carried out on the time-domain signal. We found $n_{TD}=1.606\pm 0.07$, which also fits well with the value found by transmission spectroscopy for the entire frequency range ${\bar{n}}_{transm.}=1.60$.

We find that the agreement between the two measurement techniques, reflection and transmission, is not so good for frequencies higher than 0.7 THz, despite the moderately high signal amplitude. We attribute the discrepancy to a higher ellipticity of the THz beam polarization for higher frequencies, a phenomenon already observed for PCAs with similar structures [30,31].

### 3.3. Film Thickness

In the case of thick films, if there is no overlap between the different pulses, the delay $\Delta_{t}$ can be estimated in the time domain, e.g., by measuring the distance between peaks or zero-crossings. These estimations are generally not very accurate as they ignore possible pulse distortion effects. A better method is to simulate the pulses as described in Section 2.5. In principle, this fit can be carried out with only one combination of $\left(\beta_{1},\beta_{2}\right)$, provided that the corresponding signal has a high enough amplitude and the reflection coefficients are accurately calculated. However, since the additional effort is very limited, it is advantageous to repeat the fit for a few different $\left(\beta_{1},\beta_{2}\right)$, resulting in a good signal. We do this in this work to estimate the accuracy of the method. A measurement is thus carried out in the same sequence as for the reference measurement, this time with the PVC thin film in the sample holder.

An example of an experimental pulse with sufficiently high amplitude is shown in Figure 5a (blue), together with the result from the fit using Equation (5) (red). The pulses with the highest amplitudes correspond to combinations $\left(\beta_{1},\beta_{2}\right)$ with high effective reflection coefficients amplitudes (Figure 5b). They yield the best fit quality, as can be seen from the low values of the calculated sum of squared estimate of erros (SSE) in Figure 5c. The thickness values obtained from the fits to these pulses are the ones considered to calculate the final averaged thickness value (Figure 5d). A statistical analysis of the results from Figure 5d shows that a single fit on one combination of $\left(\beta_{1},\beta_{2}\right)$ with a high amplitude gives a thickness value of d=204±6μm. The result matches well with the values found with TeraLyzer using the transmission spectroscopy, caliper and OCT method, $d_{transm.}=206\pm 7\;\mu$m, $d_{caliper}=200\;\pm \;10\;\mu$m, $d_{OCT}=202\;\pm 20\;\mu$m, respectively. Increasing the number of combinations with high amplitude, even picked randomly, allows to significantly improve the confidence interval (±1.4μm for 5 measurements, ±0.6μm for 10 measurements).

## 4. Discussion

In this paper we presented a method to determine frequency-dependent refractive index and thickness of thin film samples based on terahertz time-domain polarimetry in a reflection geometry.

The particularity of the setup resides in its rotating mounts for emitter and detector, with which the polarization angles are adjusted in a quick, precise and automated way. THz-TDS setups with varying angle for polarization-dependent measurements have been presented before, but they either rely on a rotation of the sample [32], polarization-sensitive antennas [11] or rotating polarizers [18,20,33]. The first option limits the setup configuration to a fixed combination of ($\beta_{1},\beta_{2}$), usually $\beta_{1}=\beta_{2}$ to maximize the amplitude. The second and third options leave more choices open but the amplitude of the signal is strongly diminished when the different polarization axes are not aligned. In practice, such setups only make use of one or two combinations of ($\beta_{1},\beta_{2}$). Polarizers offer a cleaner linear polarization, but often for a limited bandwidth and at the expense of amplitude loss. Alternatively, small cross-polarization components can appear without a polarizer whose effect we have already discussed in [32].

The alignment procedure of this setup as described in Section 2.2 relies on irises. Ideally, the localization and direction of the beam from the emitter would be imaged using a 2D camera, at least in first approximation. However, due to the current lack of affordable cameras for low-power THz radiation, this step has been done entirely with an arrangement of iris apertures that were temporarily added in the THz path. All things considered, the proposed setup could be upgraded with a motorized reflection guide to adjust the incident angle automatically. This would allow the measurement procedures to be carried out automatically, i.e., without intervention from an operator.

Extracting the frequency-dependent refractive index and thickness of a sample is done by identifying the Brewster angle and subsequent modeling. Experimental verification of our method was done by determining the refractive index and thickness of an unsupported single layer thin PVC film. The results for refractive index were in very good agreement with measurements done by THz-TDS in transmission and recent results by Faridi et al. [34]. In addition, the determined thickness values matched with measurements done by THz-TDS in transmission, OCT and caliper.

If the film material is unknown and needs to be identified, the measurement of the refractive index is a good indicator to determine one or several candidates based on comparison with the THz literature or a database. However, it might not be enough to identify one material with certainty. Here, the value of the refractive index and a comparison with the literature [35,36,37] would reveal several potential materials for the unknown thin film: poly(methyl methacrylate) (PMMA), polyvinylidene fluoride (PVDF), polystyrene (PS) and polyvinyl chloride (PVC). Although the latter would be the most likely candidate. An alternative spectroscopy method could in that case be employed. Here we took an ATR infrared spectrum of the sample, which showed that the thin film was pure PVC, thus confirming the results of the Brewster method.

We note that our Brewster angle analysis can in general provide not only the refraction coefficient but also the absorption of the thin film by analyzing the phase information of the reflected light or the reduction in signal amplitude after propagation through the sample [22,38]. However, it is only accurate for films with high absorption, which is not the case for the film examined in this work. In the case of low absorbing materials, the absorption coefficient can be determined by including information on the transmitted light [39,40]

We also point out that the frequency-dependent refractive index cannot be evaluated in regions of the spectrum with low amplitude such as minima stemming from Fabry–Perot oscillations. The presented method can be extended to other thin film configurations. It can be easily applied to coatings thick enough that the reflections from the individual interfaces are well separated in the time signal. If the coating is thin, the simulations have to be modified to take the different reflection coefficients from the film-coating interface into account. The proposed analysis can also be of interest for opaque films and very thick materials, as it gives access to the optical parameters of the material where a transmission measurement is not possible.

In order to reduce the cross-polarization effects at high frequencies, future developments will include the addition of broadband polarizers in the rotating PCA mounts. It has been shown that this can increase the usable bandwidth and potentially the global accuracy [31]. The method will be further advanced by testing it for coatings on a substrate and highly absorbing materials.

## 5. Conclusions

In conclusion, a method for determining the optical properties and dimensions of films using THz-TDS polarimetry in reflection geometry was put forward. The advantage of this method is that sample access is only needed from one side. Therefore, in contrast to transmission spectroscopy the film can be attached to any holder, even to non-transparent materials. Here we show its usefulness for unsupported films. Further studies are necessary to improve the instrumentation and extend our model for Brewster angle to coatings on a substrate or highly absorbing materials. Due to its advantages, this method has the potential to be applied in industry for real-time measurements of coatings.

## Abbreviations

The following abbreviations are used in this manuscript:

ATR	attenuated total reflectance
FTIR	Fourier-transformed infrared spectroscopy
OCT	optical coherence tomography
PCA	photo-conductive antenna
PMMA	poly(methyl methacrylate)
PS	polystyrene
PVC	polyvinyl chloride
PVDF	polyvinylidene fluoride
SSE	sum of squared estimate of errors
TDS	time-domain spectroscopy
THz	terahertz
UV	ultraviolet
vis	visible

## Appendix A

The detected signal depends on the PCAs’ angles and the Fresnel coefficients for a given opening of the reflection guide. The emitter and the detector rotation angles are accounted for by the following transformation matrices:

(A1) $$\begin{array}{cccc} B_{emit}\left(\beta_{1}\right) & =\left(\begin{array}{cc} \cos(\beta_{1}) & -\sin(\beta_{1})\\ \sin(\beta_{1}) & \cos(\beta_{1}) \end{array}\right) & B_{det}\left(\beta_{2}\right) & =\left(\begin{array}{cc} \cos(\beta_{2}) & -\sin(\beta_{2})\\ \sin(\beta_{2}) & \cos(\beta_{2}) \end{array}\right) \end{array}$$

The detected spectral amplitude for the reflections at the two interfaces are thus:

(A2) $$\begin{array}{cc} E_{1}\left(\nu \right) & =B_{det}\left(\begin{array}{cc} r_{s} & 0\\ 0 & r_{p} \end{array}\right)B_{emit}E_{0}=H_{S_{12}}E_{0}\left(\nu \right) \end{array}$$

(A3) $$\begin{array}{cc} E_{2}\left(\nu \right) & =B_{det}\left(\begin{array}{cc} t_{s}r_{s}t_{s} & 0\\ 0 & t_{p}r_{p}t_{p} \end{array}\right)B_{emit}E_{0}=H_{S_{21}}E_{0}\left(\nu \right) \end{array}$$

The time delay between pulses is introduced in frequency-domain by the frequency-dependent phase shift $\phi =2\pi \nu \Delta_{t}$. Absorption, which is in the present case very low, is not included in the model.

The sum over all considered reflections gives for the detected pulse in the time domain:

(A4) $$E_{det}\left(t\right)=F^{-1}\left(\sum_{k}H_{k}F\left(E_{0}\left(t\right)\right)e^{-i2\pi \nu \Delta_{t,k}}\right)$$

where F and $F^{-1}$ represent the Fourier Transform and the inverse Fourier Transform, respectively. This equation is the form used for the fit to the experimental data.
